# Possible Role of Vitamin D in Celiac Disease Onset

**DOI:** 10.3390/nu12041051

**Published:** 2020-04-10

**Authors:** Giorgia Vici, Dalia Camilletti, Valeria Polzonetti

**Affiliations:** School of Biosciences and Veterinary Medicine, University of Camerino, Via Gentile III da Varano, 62032 Camerino, Italy; giorgia.vici@unicam.it (G.V.); dalia.camilletti@unicam.it (D.C.)

**Keywords:** vitamin D, celiac disease, celiac disease onset, environmental factors

## Abstract

Beside skeletal system maintenance and protection, possible extra-calcium roles of vitamin D have been recently described. In particular, studies have investigated possible roles of vitamin D as a key modulator of inflammation and immune mechanisms and of the intestinal mucosa barrier. In this regard, vitamin D has been considered as a factor that affects different conditions such as immune-mediated diseases. The new emerging role of vitamin D and its involvement in immune modulation has led it to be considered as a possible key factor involved in celiac disease (CD) onset. CD is a chronic immune-mediated enteropathy of the small intestine that is triggered by dietary gluten protein exposure in individuals who are genetically predisposed. However, along with gluten, other environmental factors are also involved in CD onset. The renewed interest in a molecule that offers great possibilities for new roles has led to an increase in studies, although there remains a lack of studies aimed at contextualizing the role of vitamin D on CD. This review aims to define the possible role of vitamin D in CD onset as it is presently understood, taking into account potential links among vitamin D, the immune system and CD.

## 1. Introduction

Vitamin D belongs to the steroid hormone family. It has two major forms, vitamin D3 (cholecalciferol) and vitamin D2 (ergocalciferol), both of which can be found in foods or supplements, although only vitamin D3 is synthetized in skin [1]. Indeed, vitamin D3 is mainly produced endogenously in the skin by converting 7-dehydrocholesterol through the action of ultraviolet light B (UVB) of the sun or other UVB sources [2,3]. Oral intake of vitamin D is generally limited (oily fish and eggs, vitamin D fortified food), while the majority of it is derived from UVB light action. This links its content not only to dietary intake, but above all to seasonal changes, sun exposure, geographic locations and skin pigmentation [4,5]. The steroid hormone precursor vitamin D3 is synthesized within skin and is biologically inactive. Two hydroxylation reactions are required to activate it. The first one forms the 25-hydroxyvitamin D3 [25(OH)D3 or calcidiol] in the liver, then 25(OH)D3 is converted to 1,25-dihydroxyvitamin D3 [1,25(OH)2D3 or calcitriol] by the 1α-hydroxylase (CYP27B1) in the kidney. CYP27B1 enzyme is stimulated by parathyroid hormone (PTH) [4,5]. Vitamin D has been known for its role in the maintenance and safeguarding of skeletal system integrity. Indeed, biologically active vitamin D enhances calcium intestinal absorption by regulating calcium transport proteins in the small intestine, stimulating osteoclastic maturation and helping bone growth, which in turn supports collagen matrix mineralization [1,3,6,7].

The vitamin D receptor (VDR), a nuclear receptor hormone, mediates the biological activity of 1,25(OH)2D3 [4,5]. The highly polymorphic VDR belongs to the steroid receptor super family and enables vitamin D to exert its genomic actions [1,7,8].

Recently, vitamin D has received increased attention, as it was discovered that tissues and cells not involved in mineral and bone metabolism present VDR and vitamin D-activating enzymes. VDR has been reported to be expressed on cell types as antigen-presenting cells and lymphocytes, underlining a possible role of vitamin D as a key modulator of immune and inflammation mechanisms, and suggesting VDR gene polymorphisms to be markers of protection from or susceptibility to autoimmune diseases [3,7,8]. Pleiotropic actions on chronic diseases such as cardiovascular disease, diabetes, neurological disease and autoimmune disease are now under study. Over 900 genes have been reported as regulated by vitamin D [1,3]. Although its effect on the immune system and infection is an emerging topic, there has been an increased understanding related to vitamin D as a regulator and signaling component on the immune system [6,9,10].

Celiac disease (CD) is a chronic small intestinal immune-mediated enteropathy triggered by exposure to dietary gluten proteins among individuals genetically predisposed, which affects around 1% of the general population [11,12,13,14]. CD is a unique autoimmune disease for which gluten has been identified as the environmental trigger. For the disease to occur, the key genetic elements human leukocyte antigen HLA-DQ2 and HLA-DQ8, and the involvement of autoantigen (tissue transglutaminase (tTG)) are required [15,16].

The inducers of the disease are gluten and the alcohol-soluble gliadins it comprises, which are mainly present in specific cereals such as wheat, barley and rye [17].

In CD patients, the gluten contained in food products is broken down by specific gastrointestinal enzymes into peptides, which pass through the intestinal epithelial barrier to reach the mucosal lamina propria. The enzyme designated to convert gluten peptides is transglutaminase 2, which recognizes glutamine residues. Glutamine residues are converted into glutamic acid, leading the production of deamidated gluten peptides, which are now able to strongly bind to HLA-DQ2/-DQ8 molecules (the surface restriction elements for gluten-specific CD4+ T lymphocytes). Therefore, gluten peptides behave like antigens inducing an amplified immune response [18].

The cited HLA-DQ2/DQ8 molecules are the products of an expression of class II genes by the main histocompatibility complex (HLA system) [19].

Beyond genetically predisposed HLA-DQ2/DQ8 haplotypes, another characteristic in CD patients is the autoantibody response expressed against transglutaminase 2. The role of antibodies for transglutaminase 2 in the pathophysiology of CD is not clear, but their serum concentration in CD patients is increased. Autoantibodies play a role in the diagnosis of CD and in monitoring the progression of the disease after beginning a gluten-free diet, which generally reduces serum autoantibodies after a period of months [20]. 

Moreover, celiac disease patients show an altered intestinal mucosa, due to the disassembly of enterocyte tight junctions (which, in physiological conditions, contributes to making the intestinal mucosa a barrier against external agents) [21].

Tight junctions are localized in the apical part of enterocytes and are responsible for their connection. Tight junctions are made up of integral membrane proteins such as occluding, claudins and scaffolding proteins (e.g., zonulin) [22]. Thanks to their capacity to connect enterocytes, tight junctions form a barrier to regulate the transfer of molecules through the intestine. Consequently, a variation in tight junction structure can lead to increased intestinal permeability [21].

The characteristic damage that occurs during CD due to gluten exposure develops gradually [23]. The appearance of lesions characterized by intensified increased intraepithelial lymphocytes, crypt hypertrophy and progressive villous atrophy are the first typical histologic sign of CD. At a macroscopic scale, celiac disease results in malabsorption, diarrhea, bloating and (to variable degrees) undernutrition [24]. According to Livshits et al., the most widespread intestinal clinical traits are malnutrition, chronic diarrhea and nutritional deficiencies. Today, these traits are disappearing, making way for extra-intestinal presentations such as skin, endocrine, skeletal, hepatic, hematological, thrombophylic, gynecological, fertility, dental, cutaneous, neurological and behavioral abnormalities [25]. Other extra-intestinal manifestations are osteopenia, osteoporosis, fractures, arthritis and arthralgia [26]. Currently, gluten-driven symptoms and injuries are occurring at more advanced ages, and latent, hypo-symptomatic or asymptomatic manifestations are becoming amply present [27].

Diagnosing CD requires several factors: the presence of clinical signs and symptoms, seropositivity for endomysial or tissue transglutaminase autoantibodies (tTGA), the existence of HLA-DQ2/DQ8 haplotypes and, last but not least, the appearance of histologic lesions characterized by increased intraepithelial lymphocytes, crypt hypertrophy and progressive villous atrophy. However, none of these four criteria are individually sufficient to confirm CD diagnosis [28].

While gluten exposure and the presence of specific HLA antigen genotypes are necessary elements for the occurrence of CD, the disease risk is also strictly related to the timing or quantities of gluten consumed, and to the role of other potential pathophysiological factors. Therefore, the genetic predisposition is not enough on its own to be considered the triggering factor of the disease [29].

Aside from genetic predisposition, there are additional factors that play important roles in CD. Indeed, genetic background is a mandatory determinant of CD development, but environmental factors (e.g., viral infections) also contribute, such as loss of intestinal barrier function, inappropriate adaptive immune response and an imbalance in gut microbiome [16]. The role of environmental factors in relation to celiac disease onset is of great interest. In this regard, Ludvigsoon and Green have introduced the concept of a “missing environmental factor” [30].

Other than gluten, which is the most important recognized environmental trigger factor, emerging data underline the possible roles of microbiota, time of gluten introduction to children, delivery method, history of breastfeeding, acute viral gastrointestinal infections and micronutrient deficiency in the development of CD [14,31,32]. The emerging extra-calcium role of vitamin D and the increasing interest in its involvement in immune modulation led Tanpowpong and Camargo to postulate that, in genetically predisposed individuals, vitamin D deficiency can play an important role in CD onset in children. In particular, this deficiency can result in a dysregulated immune response that can contribute to an abnormal intestinal mucosa, increasing susceptibility to acute gastrointestinal infection [14]. Even if the role of vitamin D in celiac disease pathogenesis is not completely known, its potential role in immune regulation could link vitamin D deficiency to this condition, considering that vitamin and mineral deficiencies have been noted both in newly diagnosed CD patients and in CD patients with a gluten free diet (GFD) [12,14,33].

This review aims to discuss the role of vitamin D in celiac disease onset, considering key findings from literature regarding vitamin D effects on the immune system, 25(OH)D plasma levels, vitamin D supplementation both in pregnancy and in infancy and CD development.

## 2. Methods

Searches at MEDLINE/PubMed were performed in February 2020 using a combination of keywords addressing “celiac disease”, “vitamin D”, “25-hydroxyvitamin D”, “cholecarciferol”, “vitamin supplement”, “vitamin supplementation”, “pregnancy” and “children”, looking for articles published between 1 January 2010 and 7 February 2020. The following search algorithms were used:

((vitamin D) OR (vit. D) OR (25(OH)D) OR (25-hydroxyvitamin D) OR (cholecalciferol)) AND (celiac disease), which retrieved 428 papers, reduced to 213 with the “last 10 years” filter.

((vitamin D) OR (vit. D) OR (25(OH)D) OR (25-hydroxyvitamin D) OR (cholecalciferol)) AND (celiac disease) AND ((supplementation) OR (supplement)), which retrieved 80 papers, reduced to 57 with the “last 10 years” filter.

((vitamin D) OR (vit. D) OR (25(OH)D) OR (25-hydroxyvitamin D) OR (cholecalciferol)) AND (celiac disease) AND ((pregnancy) OR (pregnant)), which retrieved 21 papers, reduced to nine with the “last 10 years” filter.

((vitamin D) OR (vit. D) OR (25(OH)D) OR (25-hydroxyvitamin D) OR (cholecalciferol)) AND (celiac disease) AND ((infancy) OR (pediatric)), which retrieved 84 papers, reduced to 68 with the “last 10 years” filter.

((vitamin D) OR (vit. D) OR (25(OH)D) OR (25-hydroxyvitamin D) OR (cholecalciferol)) AND (celiac disease) AND ((molecular) OR (in vivo) OR (in vitro)), which retrieved 13 papers, reduced to six with the “last 10 years” filter.

After compiling all of the papers obtained via the research algorithms and eliminating duplicates, the full text of the remaining 44 articles were extensively reviewed on the basis of originality and relevance of each paper to the scope of this review. Thirty-five papers were selected; moreover, 25 papers were added by searching the reference lists of the 35 papers reviewed. A total of 60 articles were considered for this review.

## 3. Discussion

### 3.1. Vitamin D and Immune System

The role of vitamin D on immune function has been the subject of increasing interest, and studies have reported effects on both innate and adaptive immune responses. Vitamin D exerts effects on different aspects of immune functions, and it helps in promoting innate immunity by maintaining and improving defences against infection whilst, in parallel, regulating T-cells and dendritic cells (DCs) via a possible role in the mitigation of T-cell-mediated autoimmune disease [6,7,8,9]. The enzyme to convert 25-hydroxyvitamin D to its active form is expressed in macrophages and monocytes. It has been reported that vitamin D can participate in infection control thanks to its effect on macrophages, which enanch the antimicrobial effect. Indeed, the activation of Toll-like receptors regulates VDR expression, as bacteria lead Toll-like receptors to activate VDR expression and 25-hydroxyvitamin D-1α-hydroxylase activity to be increased, with the latter stimulating cathelicidins [6,34]. Moreover, vitamin D has shown an effect on dendritic cell activity by inhibiting monocyte differentiation into dentritic cells and decreasing IL-12 production. Effects on both B and T lymphocites have also been demonstrated. Influence on B-cell function has been reported as a consequence of differentiation and proliferation inhibition, apoptosis promotion and decreases in immunoglobulin production (autoantibodies included). It also reduces T-cell function by both decreasing T helper cell proliferation and differentiation, promoting a shift from a pro-inflammatory to a more tolerogenic immune status [6,34]. These new findings on the role of vitamin D have been underlined by studies reporting the increased risk of respiratory infection and of autoimmune disease in cases of low levels of vitamin D [9,35]. VDR is also expressed in the intestine, with a crucial role in cell proliferation, differentiation and apoptosis-induction regulation [36]. A literature revision by Masri underlined an association between vitamin D deficiency and incidences and activity of inflammatory bowel disease (IBD), with a focus on a possible role of vitamin D replacement therapy in IBD clinical practice, even if large intervention trials are needed [36]. Recently, the role of vitamin D in intestines has been described by Malaguarnera, who reported the role of vitamin D in the maintenance of gut homeostatis through a local synthesis of 1α,25(OH)2D3 and VDR expression, emphasizing that an optimal 1α,25(OH)2D3 status is fundamental, as it participates in several regulatory activities regarding not only calcium absorption but also infection protection, epithelial barrier function preservation and gut microbiota modulation. This role has received increased attention, as an unbalanced microbiota could be linked to several negative health disorders such as inflammation, allergic reactions, autoimmune diseases, heart diseases, obesity and metabolic syndrome [37].

Celiac disease is characterized by an immune response to undigested gliadin peptide fragments (e.g., pepsin–trypsin-resistant gliadin) in the small intestine. In susceptible individuals, the food-derived antigens of these fragments result in activation of the immune system and CD onset. In CD, ingested gluten leads to an immune response characterized by an interplay between innate and adaptive response. Gluten peptides are transferred to mucosal lamina propria and deamidated by tissue transglutaminase. The transfer from epithelial barrier to mucosal lamina propria could occur via either epithelial transcytosis or increased permeability of tight junctions. After the deamidation, gluten peptides bind to human leucocyte antigens (HLA-DQ2 and HLA-DQ8) on antigen-presenting cells. This step induces a response by activating CD4+ T-cells. An inflammatory response occurs, as CD4+ cells mainly secrete Th1 cytokines (as INF-gamma) that result in small bowel enteropathy, which is characterized by mucosal remodeling, villus atrophy and increasing cytotoxicity of intraepithelial lymphocytes or natural killer (NK) T-cells. Increases of intraepithelial lymphocytes and epithelial cell proliferations with crypt hyperplasia, as well as reduced enterocyte differentiation, characterize pathological lesions. In addition, CD4+ cells participate to the activation of B cells throughout Th2, resulting in the production of antibodies to gluten and transglutaminase [18,38,39,40,41].

Although the main external trigger is gluten, which is able to activate both innate and adaptive immune systems, the intestinal barrier plays a key role. Indeed, in susceptible individuals, an early disruption of the gut barrier will result in an increased permeability that could precede the onset of immune events induced by gluten [18,38].

The intestinal barrier is a complex structure that aims to prevent harmful material from passing through intestinal mucosa that could reach the lamina propria. Its dynamic interaction with environmental factors such as pollutants, microorganisms and other materials are at the base of its crucial role. Its functionality depends on several factors, including epithelial layer integrity, gut-associated lymphoid tissue and intestinal microbiota homeostasis [18,38,42].

Tight junctions (TJs) are the major junctions responsible for intestinal mucosa barrier regulation. Indeed, under physiological conditions, they limit the passage of macromolecules (including gliadin peptides) across the intestinal barrier. TJs are multiprotein junctional complexes that regulate intestinal permeability. Injuries of TJs and consequent junction disassembly are considered initial processes that lead to the entry of gliadin peptide fragments and subsequent immune response activation. In this regard, increased intestinal permeability appears to be an early biologic change that precedes the onset of autoimmune diseases, including CD [38,43,44]. 

Gliadin has been reported as causing an increase in the permeability of the intercellular tight junctions of intestinal epithelial cells. This has been linked to an enhancement in zonulin release. Zonulin is a family of molecules that is responsible for the structural disassembly of tight junctions and consequently enhancement of intestinal permeability [16].

Lammers et al. have reported that the disassembly of gliadin-induced tight junctions is caused by the binding of gliadin to chemokine receptor CXCR3 and the subsequent MyD88-dependent release of zonulin. They also underlined that CXCR3 is expressed more abundantly at the intestinal level (i.e., epithelium and lamina propria) in patients with CD than in non-CD individuals [43,44].

Fasano reported the upregulation of zonulin induced early in the disease by exposure to the disease’s antigenic trigger gliadin impacts on TJs, causing an increased opening resulting in the passage of antigens (including gliadin) [45].

Thomas et al. proposed a model in which the increased intestinal permeability caused by gluten exposure and the release of zonulin gain access to submucosa through gliadin. Gliadin stimulates macrophages in a MyD88-dependent way to promote Th1 cytokine pooling (i.e., IL-12 p70, IL-15) through up-regulation of proinflammatory gene expression and cytokine secretion [46]. The role of vitamin D is of great interest in this context, as gut epithelial cells highly express vitamin D receptor (VDR), which mediates 1,25(OH)2D3 biological activity, and immunomodulatory properties have been reported [47,48]. 

The expression and kinetics of vitamin D-related genes in human-activated T lymphocytes has been investigated by Baeke et al. They showed that 1,25(OH)2D3 effectively triggered VDR signaling, but only when introduced to T lymphocytes expressing high levels of VDR. These findings show that an enhanced degree of VDR signaling correlates with a stronger inhibition of cytokines, describing human T lymphocytes as direct targets of 1,25-dihydroxyvitamin D3 in the immune system [48].

In addition, the role of dendritic cells (DCs) in the interface between innate and adaptive immunity is of great interest in CD. Phenotype and function of DCs can be conditioned by several factors, one of which is represented by 1,25-dihydroxyvitamin D3 (1,25(OH)2D3). The potentially positive role of vitamin D on DCs has recently been underlined, demonstrating a strong relation between suboptimal vitamin D level and the occurrence and progression of many autoimmune diseases [49]. Ferreira et al. have demonstrated that in vitro treatment with 1,25-dihydroxyvitamin D3 alters the phenotype and behavior of murine bone marrow-derived dendritic cells, converting their immunogenicity into a tolerogenic profile for a reduction of T-cell responsiveness and an increased production of regulatory cytokines [50].

Another key aspect of the role of vitamin D in CD is its effect on TJ injuries.

A protective effect has been reported by several studies in different intestinal TJ injuries caused by dextran sulfate sodium and alcohol [51,52]. In the CD context, an interesting impact on TNF-α has been reported by Chen et al. They investigated the effect of 1,25(OH)2D3 on TNF-α-induced barrier dysfunction in Caco-2 cell monolayers. Their results describe a protective effect of vitamin D, which suppresses NF-kb p65 by mediating the activation of the myosin light-chain kinase (MLCK)-P-MLC signaling pathway [53]. In particular, MLCK phosphorylates MLC, which is a mediator of actin dynamics, thus leading to a contraction of the actomyosin cytoskeleton and disruption of TJs in the intestine [54].

Dong et al. demonstrated that vitamin D 1,25 had a beneficial effect on TJ injuries induced by pepsin–trypsin-resistant gliadin (PT–G) both in a Caco-2 monolayer model and in a gluten-sensitized mouse model. They reported that in all their results, VD3 was able to suppress zonulin release signaling pathway activity, upregulate TJ protein expression and attenuate increases in Caco-2 monolayer permeability. Among three different studied concentrations (10^−7^, 10^−8^ and 10^−9^ M), 10^−8^ M showed a protective effect on PT–G-induced TJ injury, underlining that it may be dose dependent. At this concentration, vitamin D was linked to an upregulation of TJ protein expression and to zonulin release suppression. Moreover, this concentration inhibited the MyD88 expression involved in zonulin release. They confirmed these findings by performing an in vivo study using gluten-sensitized mice. The mice were sensitized with PT–G for 30 consecutive days, which resulted in significant increases in small intestinal permeability, significant decreases in TJ protein expression and MyD88-dependent zonulin release signaling pathway activation. A vitamin D3 oral treatment for 7 days attenuated these changes, confirming in vivo the protective role of vitamin D against PT–G-induced intestinal mucosal barrier injuries. The authors of this study did not explore the mechanism by which vitamin D blocks the zonulin release signaling pathway, but they did hypothesize that vitamin D3 may inhibit that pathway by binding to its ligand [38].

These findings support the hypothesis that vitamin D may have a key role in CD onset by being involved both in immune response regulation (through action on dendritic cells and T-cells, above all) and on intestinal permeability by regulating inflammatory cytokines and zonulin release pathway—two key factors related to CD.

### 3.2. Celiac Disease and Enviromental Factors

The importance of defining environmental factors that are able to influence CD onset relies on the possibility of improving and studying new strategies in terms of primary prevention. In this regard, factors that are able to influence CD onset have been the subject of several studies. In particular, season of birth has become a new field of study, as a seasonally dependent fluctuation related to CD onset has been suggested (Table 1) [55,56,57,58,59]. A study conducted in Sweden showed an increased CD risk in children born during the summer compared with those born in winter. In particular, authors reported that in study group of 2151 children with verified CD, risk significantly increased in children (<2 years old) born during summer, with this seasonal pattern maintained during 10-year epidemics of CD. Moreover, it was underlined that this seasonally dependent risk decreased with age, as if this exposure only exhibited an effect in the very first year of life. Study findings suggest a possible environmental exposure characterized by seasonal patterns, and authors has hypothesized a role of viral infection or time of gluten intake [55]. In addition, Lewy et al. examined the medical records of 431 children with CD (239 girls and 192 boys), and reported a seasonal pattern in CD that was different from the general population, with peaks in September. Moreover, different seasonality was found depending on age (< or > 24 months), sex and family history of CD. The author linked study findings with perinatal virus infection as the trigger factor [56]. More recently, a case-control study conducted in Sweden using biopsy reports from 28 Swedish pathology departments with 144,522 controls matched for gender, age, calendar year and country reported an association between summer birth and increased risk of CD, but the excess risk was small and largely limited to children diagnosed before age 2. The authors of that study focused their attention on the need for further studies to test the possible mechanisms related to seasonality. In this context, they described not only the possible role infection could play in potentially increasing the risk of CD (e.g., by influencing microbiota and compromising mucosal barrier function), but also the role of low 25(OH)D levels. In particular, they considered both low 25(OH)D levels in mothers due to lack of UV light exposure during pregnancy, and in children at the time of gluten introduction and viral infection [57]. A population-based study conducted in the US analyzed CD prevalence in people living in northern latitudes compared with people living in southern latitudes. Data on gluten-related conditions were analyzed from the US National Health and Nutrition Examination Survey, from 2009 through 2014, using 22,277 participants that were 6 years and older. Authors reported a North-South gradient in CD with higher proportion on people living at latitudes of 35° North or greater independently from race or ethnicity, socioeconomic status, or body mass index. This pattern resembles the north–south gradient in the disease occurrence of autoimmune diseases, including inflammatory bowel disease, multiple sclerosis and rheumatoid arthritis [58]. In a survey conducted at two Italian centers for CD in Rome and Bari using the data of CD patients born between 2003 and 2010 were retrospectively examined to investigate whether the season of birth could be associated with CD onset in Italy. In this study, 596 children were compared, with a reference group of 439,990 controls, and data showed that children born in the summer in Italy were at higher risk of developing CD. From these findings, the authors hypothesized that there could be some factors related to a pattern of seasonality. In particular, they underlined that children born in summer are introduced to gluten during the winter, with a concomitant increased probability of rotavirus infection. Moreover, it was highlighted that 25(OH)D levels can vary during the year due to different UV exposures [59].

### 3.3. Vitamin D Status in Celiac Disease

In order to evaluate the involvment of vitamin D in CD onset, attention was focused on vitamin D status not only after CD diagnosis, but also before, especially during pregnancy and first years of life (Table 2).

In 2011, O’Malley reviewed the vitamin D statuses and supplementations in a pediatric population with gastrointestinal diseases, reporting that children with gastrointestinal disease were more likely to have a vitamin D deficiency, possibly due to malabsorption. The review also highlighted the importance of vitamin D status monitoring of children, and the need for possible supplementation in some instances [60].

The importance of introducing routine testing to evaluate vitamin deficiencies led Imam et al. to conduct a retrospective medical record review of CD patient, measuring fat-soluble vitamin levels at the time of diagnosis in order to identify the frequency of deficiencies. However, data obtained showed that fat-soluble vitamin D deficiencies were uncommon in pediatric CD, even if the study had some limitations (such as the small sample size (83 patients) and data coming from a single tertiary center) [61]. Similar results were obtained by Villanueva et al. that found no differences in vitamin D status between CD children and no-CD children in a retrospective study of 74 prepubertal children (age 3–12 years old) divided into 24 CD and 50 no-CD [62]. Further, Lerner et al. compared the vitamin D status of CD children (Israeli CD children and Spanish CD children, group 1 and group 5, respectively) to children with no-specific abominal pain (group 2), their parents (group 3) and Spanish adult CD patients (group 4). No vitamin D deficiency was found in the children. Conversely, in the adult CD population, a significant vitamin D deficiency was reported. This was explained by the authors as being related to the fact that children with CD had an increased intake in terms of vitamin D, due to the routine supplementation during the first year of their lives, as well as increased sun exposure and GFD compliance. Interestingly, they observed that vitamin D deficiency was age dependent, but this seems not to be related to degree of small bowel injury in CD. From these findings they concluded that a routine check of vitamin D status in CD patients is of fundamental importance, especially in adults [63]. A study was conducted to investigate if the prevalence of autoimmune diseases was higher in patients with CD and low vitamin D than in patients with CD but normal vitamin D. Both groups had a similar prevalence, however psoriasis risk was higher in the low vitamin D group. Although this study reported that low vitamin D was not predictive of autoimmine disease among CD individuals, it was underlined that vitamin D deficiency is common in CD, and that assessment of its values should be taken into account in clinical practice [64]. The importance of nutrient assessment in CD was also underlined by Caruso in a review that aimed to evaluate the impact of CD on iron, folate, vitamin B12, vitamin D and calcium [11]. Micronutrients are commonly below optimal levels in treated CD, and this deficiency could be related to extra-intestinal symptoms or signs [11,12].

In order to study a possible correlation among micronutrient deficiency in CD, serum tissue transglutaminase (tTG) immunoglobulin A (IgA) antibody titers and degree of mucosal damage at diagnosis, Deora et al. evaluated the prevalence of micronutrient deficiences in children both at diagnosis and at 6 and 18 months after the start of a GFD. Examining the medical records of 140 children with CD, a 70% deficit in vitamin D was found. In addition to this, the data showed no correlation between degree of villous atrophy or serum titers of anti-tTG IgA molecules and micronutrient deficiency. Moreover, almost all micronutrient deficiences found at the diagnosis were reported to have normalized after 6 and 10 months of a GFD. Only vitamin D and ferritin still remain under optimal levels after 18 months, highlighting the importance of their long-term assessments. In this regard, the authors of the study reported that vitamin D status evaluation should be performed annually in children [65].

As nutritional deficiences are commonly found in gastointestinal disease, Ahlavat et al. reviewed the prevalence of vitamin D deficiency in pediatric gastrointestinal disease. They reported that even if the exact role of vitamin D is not fully understood, the actual knownledge in bone health and the role in immune regulation mean that determining vitamin D status is very important to screening for diseases such as IBD and celiac diasease. From a practical point of view, they screened for low vitamin D status at disease onset and, if needed, treated for any level greater than 30 ng/mL but less than 100 ng/mL. However, large randomized controlled trials are needed in order to further investigate the role of vitamin D in disease remission and the maintenance of optimal 25(OH)D levels [33]. Recently, a cross-sectional study conducted with the aim of screening for vitamin D status reported no significant difference between newly diagnosed CD patients and no-CD controls in regards to vitamin D status. However, inadequante 25(OH)D levels were found in both groups. Moreover, vitamin D levels were highly associated with estimated vitamin D intake in both the CD and control groups. Form these findings, the authors recommended a vitamin D status screening in children at diagnosis time [66].

The impact of vitamin D status on CD onset has also been investigated in pregnant women, as low concentrations of it have been associated with offspring autoimmune disease. In particular, Marild et al. tested whether low maternal and neonatal 25(OH)D levels could be a predictor of increased CD risk in a cohort of pregnant norwegian women. In particular, mid-pregnancy and post-partum blood analysis and cord plasma were performed, comparing data of 416 children who developed CD and 570 children without nCD. In addition to this, mothers and children were genotyped for established celiac disease and vitamin D metabolism variants. Obtained results showed no significant difference of 25(OH)D levels between the two group, and genetic variants for deficiency did not associate with CD. The authors concluded that even if they could not exclude the presence of unmeasured confounders, level of 25(OH)D seems not to be associated with pediatric CD onset. However, randomized controlled trials will be needed to further investigate this topic [9]. In 2019, a cross-sectional study was conducted to evaluate whether severe vitamin D deficiency is assosiated with CD by evaluating 200 Saudi adolescent girls. Data obtained showed CD as a potential a risk for severe vitamin D deficiency and, above all, that low levels of vitamin D (<12.5 nmol/L), in absence of obvious causes, could be a sign of the need for CD screening [67].

In terms of supplementation, Bittker reported that oral vitamin D consumption among children could be a significant risk factor in inducing CD. Bittker related this to the fact that vitamin D tends to elevate the activity of Th2 cytokine, which upregulates immune reactions to external stimuli, reporting that vitamin D has been described as a risk factor for allergic diseases including asthma and atopic dermatitis, with high comorbidity with CD [68]. Recently, the same author reported that the “missing environmental factor” could be significant oral vitamin D exposure with its consequent increase in plasma. Oral vitamin D in large doses upregulates cytokines, chemokines and Toll-like receptors that are also upregulated in CD. Moreover, epidemiologically higher CD prevalence has been reported in countries with aggressive supplementation policies [69]. As vitamin D could be considered an immunomodulator, and its role in CD has been recieved increased importance, Bittker conducted a case-control epidemiological survey among parents living in the US with at least one biological child between 3 and 12 years old to determine if nine varibales could be associated with CD in children. Among these, vitamin D drop exposure in infancy and vitamin supplement exposure between 2–3 years were evaluated. Data obtained from 332 children diagnosed with CD (cases) and 241 without CD (controls) suggest that infants who received oral vitamin D drops for longer than 3 months were at increased risk of subsequently developing CD [70]. On the contrary, a longitudinal prospective observational study on 6627 children reported that maternal use of dietary supplements during pregnancy is not associated with CD. In particular, maternal use of vitamin D, *n*-3 fatty acids and iron were not associated with CD risk, concluding that dietary supplementation during pregnancy could be a tool to improve nutrient intake [71].

### 3.4. Vitamin D and International Guidelines

Even if the interest in the role of vitamin D in CD is increasing, previously reported data have been controversial, and specific studies and indications regarding optimal serum levels and supplementation strategies are still lacking. Controversy remians regarding the optimal level of serum vitamin D, with governamental agencies reporting sufficiency at levels of 25(OH)D >20 ng/mL (> 50 nmol/L). However, evidence related to both skeletal and non-skeletal outcomes has defined the vitamin D deficiency threshold as 30 nmol/L [66,72,73,74].

The American College of Gastroenterology reccomended the assessment of micronutrient deficiences for, but not limited to, iron, folic acid, vitamin D and Vitamin B12 in newly diagnosed CD [75]. The British Society of Gastroenterology (BSG) CD guidelines suggest measuring calcium, alkaline phosphatase, vitamin D levels and parathyroid hormone both at diagnosis and when neccessery [76]. Specifically for pediatric patients, the North American Society for Pediatric Gastroenterology, Hepatology and Nutrition (NASPGHAN) reccomends vitamin D assessment at diagnosis and annually [77]. An Italian consensus suggests serum vitamin D levels be evaluated in CD patients at diagnosis and after 6–12 months of GFD if deficiency has been found [78]. A panel of experts published reccomendations for managing CD in children, and suggested screening vitamin D at diagnosis; however, the quality of evidence was considered low and the strenght weak. They suggested considering age-appropriate counseling regarding calcium and vitamin D supplementation at diagnosis and during follow up (evidence grade high, statement strength strong) [79]. The Endocrine Society Clinical Practice Guideline advises evaluating vitamin D status in patients at risk of deficiency, suggesting supplementation either with vitamin D2 or D3 for deficient patients [72].

Recommendations from the National Institute for Health and Care Excellence (NICE) on the recognition, assessment and management of celiac disease based on systematic reviews of available evidence suggests the possible needs of vitamin D supplementation in cases of insufficient dietary intake. However, they reported very low-quality randomized controlled trials the basis of this recommendation [80].

Governmental agencies, including the Institute of Medicine (IOM) in North America and the European Food Safety Authority (EFSA) in the EU, have set the estimated average daily intake of vitamin D for healthy adults (with minimal or no sunlight exposure) at 400 IU per day [81,82]. In particular, IOM stated that for vitamin D (assuming minimal sun exposure), the estimated average requirement (EAR) is 400 IU/day for ages older than 1 year, and the recommended dietary allowance (RDA) is 600 IU/day for ages 1 to 70 years and 800 IU/day for 71 years and older. The tolerable upper intake level (UL) ranges from 1000 to 4000 IU daily for vitamin D: 1000 IU for infants 0–6 months, 1500 IU infants 6–12 months, 2500 IU for children 1–3 years, 3000 IU for children 4–8 years and 4000 IU for children 9 years and older [81]. An EFSA panel set the adequante intake (AI) for adults at 15 µg/day, for children aged 1–17 years at 15 µg/day and for infants aged 7–11 months at 10 µg/day. For pregnant and lactating women, the panel set the same AI as for non-pregnant non-lactating women, (i.e., 15 µg/day). The UL for adults (including pregnant and lactating women) was set at 100 µg/day. In children and adolescents aged 11–17 years, the UL was adapted to 100 µg/day. For children aged 1–10 years a UL of 50 µg/day was proposed, and for infants it was set at 25 µg/day. A subsequent update set the upper limit at 25 µg/day for infants aged up to 6 months, but a UL of 35 µg/day for infants 6–12 months [82,83,84].

However, specific guidelines and indications relating to both optimal vitamin D status and supplementation specifically related to CD in terms of primary and secondary prevention are still lacking. More studies, and randomized controlled trials in particular, are needed to further and better evaluate the role of vitamin D in CD onset and during the follow up.

## 4. Conclusions

Considering the importance of environmental factors in CD pathophysiology, several aspects are currently under study in order to find a possible association with CD onset. Presently, one important focus of attention relies on birth seasonality, as CD risk seems to increase in infants born during the summer. This aspect could be related to other factors, such as time of gluten ingestion, virus infections, different UV exposures or mother’s vitamin D status during pregnancy, as well as a concomitant presence of these factors. In particular, the emerging extra-calcium role of vitamin D and the increasing interest on its impact on the immune system and intestinal barrier permeability leads to its consideration as an important factor possibly involved in CD onset.

However, there have only been a few fragmentary studies, with different focuses and studied populations. Moreover, the role of supplementation is still uncertain. It is common to routinely consider vitamin D supplementation; however, this has sometimes been considered ineffective.

In light of this, clarifying the role of vitamin D is of great importance. There is a need for larger studies that would take into account variables that can have a role in CD onset (i.e., vitamin D levels of pregnant women, vitamin D supplementation and/or UV exposure), as well as involving different countries. This would be of fundamental importance in order to better explore possible associations and, in particular, to study and evaluate the possible role of vitamin D that could therefore play a key role in terms of prevention and be part of possible new CD prevention strategies.

## Figures and Tables

**Table 1:** Seasonally dependent fluctuation related to celiac disease (CD) onset.

	Country and Year	Design	Study Population	Method	Outcomes
**Ivarsson et al.** [55]	Sweden, 2003	Retrospective and prospective study	2151 CD children below 15 years of age.	CD incidence rates were calculated by month of birth, stratified for age at diagnosis.	The risk for celiac disease was significantly higher if born during summer compared to winter in children below 2 years of age at diagnosis. This relative seasonal risk pattern prevailed during a 10-year epidemic of celiac disease, although incidence rates varied threefold. The incidence was constantly higher among girls, but boys showed a more pronounced seasonal variation in risk.
**Lewy et al.** [56]	Israel, 2009	Retrospective study	431 CD children (239 girls, 192 boys). 138 girls and 81 boys were under the age of 24 months.	Medical records were analyzed to obtain both statistical significance and parameters of rhythms.	Boys and girls with CD were found to have different seasonality of month of birth. Girls diagnosed before age 24 months (peak July–August) had a different seasonality birth from those diagnosed after age 24 months (no rhythm) and showed a different seasonality from boys with diagnosis above 24 months (peak July). Different seasonality was found in children with a family history of CD.
**Lebwohl et al.** [57]	USA, 2013	Case-control study	351,403 biopsy reports, of which 29,096 from patients with CD. Up to 5 controls for each CD individual were identified by Statistics Sweden (total: 144,522).	The association between summer birth (March–August) and later CD diagnosis was examined through conditional logistic regression.	54.10% of individuals with CD vs 52.75% of controls were born in the summer months. Summer birth associated with a small increased risk of later CD. Stratifying CD patients according to age at diagnosis, the highest OR was found in those diagnosed before age 2 years, while summer birth was not associated with a CD diagnosis in later childhood (age 2–18 years), but had a marginal effect on the risk of CD in adulthood.
**Unalp-Arida et al.** [58]	USA, 2017	Population-based study	22,277 participants 6 years and older.	Analyzed data on gluten-related conditions from the US National Health and Nutrition Examination Survey, from 2009 through 2014 identifying persons with CD.	0.7% of participants were found to have CD. Celiac disease was more common among individuals who lived at latitudes of 35–39° North or at latitudes of 40° North or more than individuals who lived at latitudes below 35° North, independent of race or ethnicity, socioeconomic status or body mass index.
**Capriati et al.** [59]	Italy, 2015	Retrospective study	596 CD patients children (age range 3.5) compared with 439,990 controls	Survey conducted at two Italian referral centers for CD in Rome and Bari. The CD database was created to enable retrospective examination of the data of all the consecutive patients born between 2003 and 2010 who had received a diagnosis of CD.	A summer birth preponderance was observed in CD patients compared to controls. Stratifying the case by gender, the summer birth preponderance was maintained for females.

CD = celiac disease; OR = odds ratio.

**Table 2:** Vitamin D status in celiac disease.

	Country and Year	Design	Study Population	Methods	Vit. D Supplement	Outcomes
**Imam et al.** [61]	USA, 2014	Retrospective study	83 CD patients: 51 girls and 32 boys (average age at diagnosis, 12.8 and 13.0, respectively)	Medical record review of CD patients and fat-soluble vitamin levels measured at diagnosis between 1995 and 2012 at Mayo Clinic.	None receiving vitamin supplements at the time of diagnosis.	Average 25(OH)D vitamin levels 32.8 ng/mL; 9 patients had mild-to-moderate vitamin D deficiency, 31 patients showed insufficiency of 25(OH)D.
**Villanueva et al.** [62]	USA, 2012	Retrospective study	24 prepubertal CD children and 50 no-CD (age, 3–12 years)	25(OH)D level measured by chemiluminescence immunoassay. Height and body weight measured to calculate BMI.	No supplementation.	No difference in 25(OH)D level between CD and no-CD. Non-obese CD had a significantly higher 25(OH)D level than the obese no-CD. No difference in 25(OH)D level in non-obese.
**Lerner et al.** [63]	Israel and Spain, 2012	Case-control study	272 individuals in five groups. Group 1: 51 Israeli children with CD (age, 6 ± 4 years); Group 5: 59 Spanish children with CD (age, 4 ± 4 years); Group 2: 56 Israeli children with nonspecific abdominal pain (age, 8 ± 5 years); Group 3: 84 adults, parents of group 2 (age, 39 ± 8 years); Group 4: 22 Spanish adults with CD (age, 44 ± 13 years).	Vitamin D serum levels investigated in CD populations compared to children with nonspecific abdominal pain, their parents and Spanish adult CD patients. 25(OH)D checked by chemiluminescent immunoassays.	No supplementation.	Groups 5 and 1 had the highest levels compared to groups 4 and 3. Levels in groups 1 and 2 were comparable. Concerning 25(OH)D sera levels, only the difference between groups 5 and 4 was statistically significant. Vitamin D sera levels negatively correlated with age.
**Tavakkoli et al.** [64]	USA, 2013	Retrospective cross-sectional study	530 CD adult patients	Compared patients with normal vitamin D level (≥30 mg/dL) against those with vitamin D insufficiency (20–29 mg/dL) and vitamin D deficiency (<20 mg/dL) with regards to prevalence of autoimmune disorders.	Patients were not excluded from the study if they were taking vitamin D supplements, but there was no knowledge of supplementation.	25% showed vitamin D deficiency. Similar prevalence of AD among those with normal vitamin D (11%), insufficiency (9%) and deficiency (12%). Vitamin D deficiency was not associated with AD. Risk of psoriasis was higher in patients with vitamin D deficiency.
**Deora et al.** [65]	Canada, 2017	Single-center cohort study	Medical records of 140 CD children (mean age at diagnosis 7.8 ± 4.01 years)	Analysis of the medical records of all children with CD. Routine celiac blood tests carried out at diagnosis 6 months after starting GFD, then on an annual basis. Histopathological changes of duodenal biopsies at diagnosis documented using modified MARSH classification. Diet assessment with proper teaching of GFD at diagnosis, 6 months and 18 months after diagnosis.	During each clinic visit, an experienced dietitian evaluated the nutritional status and need for micronutrient supplementations.	70% of subjects with serum vitamin D deficiency. No correlation between micronutrient deficiencies at diagnosis and serum tTG IgA antibody titers or the degree of villous atrophy. The majority of serum levels of micronutrients normalized after 6 months after beginning GFD, except for vitamin D, which improved but remained subnormal.
**Ahlawat et al.** [66]	USA, 2019	Cross-sectional study	38 newly diagnosed CD patients (10.4 ± 3.0 years old) and 82 controls (11.2 ± 4.2 years old).	25(OH)D levels drawn in children with newly diagnosed CD compared with pediatric outpatients with functional abdominal complaints. Anthropometric data and vitamin D intake based on milk and multivitamin ingestion were recorded.	Patients were excluded if they used single-preparation vitamin D supplements within 3 months of study enrollment. Multivitamin use was allowed.	Both groups were similar except for average daily D intake and BMI. No statistical difference in mean 25(OH)D levels between CD and controls. Both groups had high percentages of suboptimal D status. 25(OH)D levels significantly correlated with age (*r* = −0.262) and estimated vitamin D intake (*r* = 0.361).
**Mårild et al.** [9]	Norwey, 2017	Case-control study	416 children who developed celiac disease, 570 randomly selected controls and their mothers	25(OH)D examined in maternal blood from mid-pregnancy, post-partum and cord plasma. Mothers and children genotyped for established celiac disease and vitamin D metabolism variants.	Participants completed a food frequency questionnaire covering the period from start of pregnancy until completion around week 22, to evaluate their vitamin D intake while also considering supplements.	No significant difference in average 25(OH)D between cases and controls and no significant linear trend. Genetic variants for vitamin D deficiency were not associated with CD.
**Sulimani** [67]	Saudi Arabia, 2019	Cross-sectional study	200 adolescent females aged 13–19 years old with vitamin D deficiency	Female adolescent screened for IgA tTG (anti-tissue transglutaminase antibodies).	No vitamin D supplement.	Of the 200 girls, 9 (4.5%) were positive for IgA tTG antibodies; all of whom had serum 25(OH)D < 12.5 nmol/L. A strong significant inverse association was found between tTG antibody levels and serum 25(OH)D (*R* = −0.53) among antibody negative participants. Positive IgA tTG antibodies were 37.2 times higher among participants with 25(OH)D < 12.5 nmol/L than those whose vitamin D status was higher.
**Bittker** [70]	USA, 2019	Case-control study	332 parents with CD children + 241 parents with no-CD children (controls)	An Internet-based survey was conducted among parents living in the US with at least one biological child between 3 and 12 years old in order to determine if 9 variables are associated with CD, among these: vitamin D drop exposure in infancy and vitamin D supplement exposure between 2–3 years old.	Two questions examined supplemental vitamin D exposure. One focused on the duration of exposure to vitamin D drops in infancy. The other on vitamin D supplementation between 2–3 years of age.	In this dataset, only vitamin D drops administered for more than 3 months was associated with CD children.
**Yang et al.** [71]	USA, Finland, German, Sweden, 2017	Longitudinal prospective observational study	6627 children (years range 0.9–10.0)	Examined the association between maternal use of vitamin D, *n*-3 fatty acids (FA) and Fe supplements during pregnancy and risk for CD autoimmunity (CDA) and CD in the offspring.	Use of supplements containing vitamin D, *n*-3 FA and Fe was recalled by 66%, 17% and 94% of mothers, respectively, at 3–4 months post-partum. The mean cumulative intake over the entire pregnancy was 2014 μg vitamin D (SD 2045 μg), 111 g *n*-3 FA (SD 303 g) and 8806 mg Fe (SD 7017 mg).	Of 6627 enrolled children, 1136 developed CDA at a median 3.1 years of age (range 0.9–10) and 409 developed CD at a median 3.9 years of age (range 1.2–11). No statistically significant association between the intake of vitamin D, *n*-3 FA or Fe, and risk for CDA or CD. Dietary supplementation during pregnancy did not seem to modify the risk for the disease in the offspring.

AD = autoimmune disorder; BMI = body mass index; CD = celiac disease; CDA = celiac disease autoimmunity; FA = fatty acids; GFD = gluten free diet; tTG = anti-tissue transglutaminase; 25(OH)D = 25-hydroxyvitamin D.
